# Polytraumatization, defense mechanisms, PTSD and complex PTSD in Indian adolescents: a mediation model

**DOI:** 10.1186/s40359-023-01456-0

**Published:** 2023-11-24

**Authors:** Paulo Ferrajão, Carolina Isabel Batista, Ask Elklit

**Affiliations:** 1 Faculdade de Ciências Sociais e Tecnologia, Universidade Europeia, Quinta do Bom Nome, Estrada da Correia 53, Lisbon, 1500-210 Portugal; 2 National Center for Psychotraumatology, University of Southern Denmark, Odense, Denmark

**Keywords:** Adverse childhood experiences, Emotion regulation, Defense mechanisms, Structural equation modelling, Adolescents

## Abstract

**Background:**

Adolescence is recognized as a particularly susceptible developmental period for experiencing multiple types of Adverse Childhood Experiences (ACE), increasing the vulnerability to higher levels of Post-Traumatic Stress Disorder (PTSD) and Complex PTSD symptoms. Some studies found that defense mechanisms play an important role on the association between ACE and psychological symptoms.

**Methods:**

We analyzed the associations between direct and indirect exposure to ACE and PTSD and Complex PTSD (affective dysregulation, negative self-concept and disturbances in relationships) through the mediation role of mature defense mechanisms: mature, neurotic, and immature defense mechanisms in Indian adolescents. A sample of 411 Indian adolescents (M = 14.2 years old; S.D. = 0.5) completed validated self-report questionnaires. Serial multiple mediation models were tested by conducting a structural equation modelling employing Preacher and Hayes’ procedures (2008).

**Results:**

Immature and neurotic defense mechanisms mediated the association between direct exposure to ACE with PTSD symptoms. Immature defense mechanisms were mediators of the relationship between direct exposure to ACE and Complex PTSD symptoms clusters.

**Conclusions:**

Maladaptive defense mechanisms can disturb the process of self-regulation and emotion regulation capabilities in coping with traumatic experiences, leading to higher PTSD and Complex PTSD symptoms severity.

## Background

Adverse Childhood Experiences (ACE) are conceptualized as traumatic or adverse events, prior to the age of 18 [1]. ACE involve two main harmful experiences: maltreatment (e.g., physical abuse and emotional neglect), and exposure to household dysfunction (e.g., parental loss and death of a family member) [2]. Recent research has expanded on the concept, adding childhood adversities that occur outside the home, namely bullying and exposure to community and collective violence [3]. ACE strongly predict negative mental health outcomes in adulthood, namely substance abuse, depression, and suicide [4, 5].

Exposure to ACE can occur directly and/or indirectly. Direct exposure encompasses first-hand exposure to traumatic or adverse experiences. Indirect exposure involves the experience of witnessing another person’s experience of violence or adversity, either firsthand or through a narrator’s description of the event [6]. Prior findings noticed differential mental health consequences associated with direct and indirect ACE exposure. Specifically, direct exposure was associated with both depression and Post-Traumatic Stress Symptoms (PTSS) severity, whereas indirect exposure was associated with anxiety and PTSS severity [7, 8].

Meanwhile, a single trauma model approach has been historically the trend by exclusively analyzing the impact of single ACE on individuals’ mental health. However, previous studies indicate that exposure to one type of ACE increases the risk of being exposed to other types of adverse experiences [6, 9]. The experience of a wide range of multiple traumas is defined in literature as polytraumatization [9], which has a more negative impact on one’s physical and mental health, namely Post-Traumatic Stress Disorder (PTSD) [10, 11], depression and dissociation [12], particularly when compared to single-trauma exposure.

In this matter, adolescence is recognized as a particularly susceptible developmental period for experiencing multiple types of ACE [13, 14]. This higher risk of exposure to ACE could be explained by developmental changes that occur in this critical transitional period, namely biological, psychological, and social changes that potentially increase the occurrence of high-risk behaviors (e.g., self-injurious behavior, unsafe sexual behaviors and/or substance abuse), thereby assuming itself as a particularly vulnerable period to polytraumatization among many adolescents [15].

Most studies have been conducted in western adolescents’ samples. However, the risk of exposure to ACE appears to be higher in non-western countries, such as India. India has a unique and complex socioeconomic and cultural context that constitutes a risk to the experience of adversity, namely extreme poverty, poor healthcare, cultural beliefs in harsh discipline, gender-based inequalities and violence, terrorism, and political conflict [16, 17]. It is reasonable to assume that each of these circumstances enhance the likelihood of Indian adolescents suffering from higher exposure to multiple ACE when compared to western adolescents.

Previous studies noticed two out of every three children have experienced physical abuse and half have experienced sexual and emotional abuse [18], and 78.1% of the sample experienced at least one potentially traumatizing event, directly or indirectly, in a study with youth from Pune City [17]. To the best of our knowledge, only a few studies on psychological consequences of exposure to ACE have been undertaken in Indian samples, and they have largely focused on specific, singular trauma events [19–21]. Therefore, there is a need to better understand the effects of polytraumatization among Indian adolescents.

It is well established that individuals who have experienced and/or were exposed to ACE show higher severity of both PTSS [22, 23] and Complex Post-Traumatic Stress Disorder (CPTSD) symptoms (CPTSDS) [24–26] in adolescence and young adulthood. The International Classification of Diseases-11 (ICD-11) [27] added a new PTSD syndrome referred as CPTSD. The ICD-11 model of PTSD includes three symptom clusters: reexperience of the trauma, avoidance of traumatic reminders, and sense of threat. CPTSD includes three additional symptoms clusters: affective dysregulation, negative self-concept, and disturbances in relationships. These additional symptoms clusters are commonly referred to Disturbances in Self-Organization (DSO) [28].

Even though several studies have evidenced the link between ACE with both PTSS and CPTSDS [29, 30], recent studies observed that this relationship is mediated by several psychosocial variables [29, 31, 32]. Therefore, it is important to better understand the psychological factors underlying the association between multiple exposure to ACE with PTSS and CPTSDS to design tailored psychological intervention in adolescence.

The capacity for self-regulation and interpersonal regulation have been proposed as crucial to prevent psychological disturbances in individuals exposed to multiple ACE [33]. Literature mostly focused on explicit emotion regulation (conscious strategies to deal with distressing experiences). However, emotion regulation also involves implicit emotion regulation (unconscious cognitive and affective processes), aimed at modifying the intensity and/or duration of emotional responses associated with adverse experiences [34]. The latter has been found to be more important in protecting children exposed to ACE from psychopathology than the former [35, 36].

Implicit emotion regulation is inherently linked to defense mechanisms (DM) [37]. Like implicit emotion regulation, DM are automatic and unconscious processes intended to protect the individual against internal or external threats and unpleasant emotions with the goal of reducing psychological distress [38]. DM are recognized for its clinical utility and the predictive value for positive adaptation is profound [39]. The association between the exposure to ACE and DM has recently received growing interest in empirical research.

DM have been defined as a continuum according to their level of maturity and adaptiveness into three levels: immature (IDM), neurotic (NDM) and mature (MDM). IDM (e.g., splitting, dissociation) aim to inhibit the awareness of the internal and/or internal experience by maintaining an illusion of emotional control through distortion of the source and importance of stress. NDM (e.g., repression and reactive formation) intend to keep potentially threatening feelings, ideas, memories or fears out of awareness. Finally, MDM (e.g., sublimation and suppression) enhances gratification, preserving a relatively more conscious awareness of feelings, ideas, and their consequences [39, 40].

Childhood and adolescence are critical developmental periods in the formation of DM [40]. In children and adolescents exposed to multiple ACE, DM play a key role in the regulation of emotions and turmoil associated with traumatic experiences and management of inevitable losses [41, 42]. Specifically, DM are used to hide or lessen internal and/or external distress associated with polytraumatization [43], mediating one’s individual reaction to traumatic and adverse experiences [37, 44].

Exposure to ACE may impact defensive functioning involving a regression from mature to survival-serving defenses to cope with multiple adversity [45, 46]. In accordance, prior research found that higher exposure to ACE was associated with higher levels of both IDM and NDM [47]. However, other studies found that higher exposure to childhood trauma only increased levels of IDM [8, 48]. On the contrary, higher exposure to ACE has been found to be associated with lower levels of MDM in a clinical sample [49].

Previous research on adolescent samples also underscores the role of DM in psychosocial adjustment. It was observed lower use of IDM and higher use of MDM in nonclinical adolescents compared with adolescents presenting psychosocial adjustment problems [50, 51]. Likewise, higher psychological symptoms severity was positively associated with levels of IDM and NDM, and negatively associated with levels of MDM in nonclinical samples of adolescents [8, 52–54].

The impact of DM on psychological symptoms in polytraumatized individuals has been observed in previous research. Higher levels of IDM are associated with increased psychological symptoms severity among individuals exposed to ACE [8, 55–57]. Conversely, higher levels of MDM are correlated with lower psychological distress [14]. Higher levels of IDM were found in individuals with a diagnosis of PTSD following exposure to war related trauma [39, 56] and clinical patients diagnosed with CPTSD [58].

Most of the research has focused on the direct association between DM and psychosocial variables, without considering the role of DM in protecting the individual against exposure to threatening and dangerous events [46]. The findings described above suggest that DM may mediate the association between exposure to ACE and psychological symptoms in polytraumatized individuals. Some studies found that IDM mediated the relationship between exposure to ACE and psychological symptoms severity in adults [57, 59]. In adolescent samples, it was found that IDM mediated the associations between direct exposure to ACE with both anxiety and depression symptoms [8].

It can be proposed that exposure to ACE is associated with a low level of MDM and a high level of IDM. This may relate to a regression of mature defenses into an earlier stage, or through revitalizing the deployment of simple, immature, and survival-serving defenses [8, 46]. Specifically, higher use of IDM may be an attempt to deal with the context of repeated or multiple traumatization through escape or avoidance of unwanted feelings associated with violent and adverse experiences by distorting or refusing the acknowledgement of personal losses and/or stressors, while creating an illusion of control over the generalized violence and adversity [43, 60].

There is a strong need for studies on the relationship between DM and psychosocial variables, such as PTSS and CPTSD symptoms in adolescents. Considering the findings presented above, it is reasonable to speculate that the development of PTSS and CPTSD symptoms in a context of multiple exposure to ACE may be mediated by specific DM. However, to the best of our knowledge, no previous study has investigated whether DM mediate the impact of multiple exposure to ACE on both PTSS and CPTSD symptoms in a sample of Indian adolescents. Based on previous findings described above, the following hypothesis were examined:

**H1**

Higher direct exposure to ACE is associated with higher levels of PTSS and CPTSDS clusters;

**H2**

Higher indirect exposure to ACE is associated with higher levels of PTSS;

**H3**

Higher direct and indirect exposure to ACE is associated with higher levels of IDM and NDM, and lower levels of MDM;

**H4**

Higher levels of IDM are associated with higher levels of PTSS and CPTSDS clusters, and higher levels of MDM are associated with lower levels of PTSS and CPTSDS;

**H5**

Serial multiple mediation models will indicate that direct and indirect exposure to multiple ACE are associated with higher levels of IDM, that will relate to higher levels of PTSS and CPTSDS clusters.

## Methods

### Participants

A sample of 411 adolescents participated in this study. Sample characteristics are presented in Table 1. The mean age of the sample was around 14 years old (age range: 13–16 years old). The proportion of males (53.3%) was higher compared with females (46.7%). Almost all participants lived with both parents. Most adolescents’ fathers completed university level, and most of the adolescents’ mothers completed upper secondary school/business school or vocational or medium cycle higher education.

**Table 1:** Sample demographic characteristics

	Total (N = 411)
**Age**	
13 years	17 (4.1%)
14 years	316 (76.9%)
15 years	77 (18.7%)
16 years	1 (0.2%)
Mean	14.2 (SD = 0.5)
**Living with**	
Both parents	395 (96.1%)
One of their parents	13 (3.2%)
Other arrangements (uncles, siblings, grandparents or other relatives)	3 (0.7%)
**Father education**	
Less than 9 years	8 (1.9%)
Lower secondary school (9 years)	6 (1.5%)
Upper secondary school/business school (10–12 years)	27 (6.6%)
Vocational or medium cycle higher education (13–15 years)	144 (35.0%)
University level	226 (55.0%)
**Mother education**	
Less than 9 years	12 (2.9%)
Lower secondary school (9 years)	142 (20.7%)
Upper secondary school/business school (10–12 years)	40 (9.7%)
Vocational or medium cycle higher education (13–15 years)	182 (44.3%)
University level	169 (41.1%)

### Procedure

The primary aim of the current study was to collect data about previous trauma exposure and trauma reactions among Indian adolescents. The Institutional Review Board of Aarhus University approved the study. The third author of the manuscript was employed by Aarhus University at the time when the study was planned, and data collected. The adolescents who participated in the study were from the city of Pune in the state of Maharashtra. Data collection was only conducted in Pune due to limited resources such as time and finances, and only students from private schools were selected for the study. Prior to data collection, invitations to participate in the study were sent to five schools on a convenience basis, but only two decided to enroll in the study. The students were primarily from a middle- and upper-class background. Each of the eight classes of students who participated in the study consisted of 50 to 60 adolescents.

The questionnaire and a letter explaining the aims of the study were introduced to the headmasters and the boards of the schools which reviewed and approved the study. A pilot study was first conducted with seven respondents at the age of 13–14 years. Most school studies in the middle-and high-income countries apply passive consent, i.e., the parents are informed about the study and have the right to refuse the participation of their child. In India, the parents trust the school system and the teachers who are in parentis loco, i.e., they are granted the position to act in the best interests of their children.

Information about the study aims, procedures and the role of the participant was introduced to the students verbally and by letter. The participation was voluntary and those accepting to participate, gave their informed consent directly. The students filled in the questionnaire in the classroom, supervised by a team researcher in co-operation with two Hindi speaking Indian psychology students, who explained the purpose of the study, the principles of confidentiality and practicalities in answering the questionnaire. The students were informed that their answers were anonymous, and they were asked to answer as openly as possible, despite the somewhat uncomfortable subject. All students present accepted to participate in the study.

The researcher requested that the headmaster of all three schools would spare one or more teachers for each class. The students did not seem uncomfortable answering the questions and the teacher encouraged them to answer honestly and tell everything. The teachers are the steady figures in the lives of the children and available to them on a daily basis. Besides their teaching, they are also aware of the plight of their students and react if they observe them to be distressed. There were no psychosocial services available in the rural district. The study contributes to the future establishment of psychosocial services in the district and therefore is in the best interest of the children if any of them were distressed, even if only temporarily. This is an accepted scientific value that contributes to the wellbeing of the children.

### Measures

*Sociodemographic data*. Participants provided information on their sex, age, highest level of parental education and current living arrangements.

*Potentially Traumatic Events*. It was asked to participants if they had been exposed directly and/or indirectly to a list of 20 life-threatening experiences (e.g., rape) and stressful family conditions (e.g., neglect). The measure was developed by Bödvarsdóttir and Elklit [61] who selected the list of events from scientific literature and clinical experience. This measure has been widely applied cross-culturally [62].

*Defensive Style Questionnaire*. The Defensive Style Questionnaire (DSQ-40) [63] assessed DM divided into 3 groups of factors: MDM, NMD, and IDM. This measure includes 40 self-report questions answered on a 9-point Likert scale (where “1” indicates “completely disagree” and “9” indicates “fully agree”). In this study, the total scores on the MDM, NDM, and IDM were analyzed. The reliability of the MDM scale (α = 0.79), the NDM scale (α = 0.82), and the IDM scale (α = 0.85) were good.

*PTSD Item Set*. Following Elklit et al.’s procedure [64], six items were selected from the Harvard Trauma Questionnaire: Part IV (HTQ-IV) [65] to assess PTSS, answered on a 4-point Likert scale (from “not present” = 1, to “very often present” = 4). The items representing PTSS are shown in Table 2. The reliability of the item set (α = 0.90) was good.

*CPTSD Item Set*. Six items were selected from two standardized measures, the HTQ-IV and the Trauma Symptom Checklist (TSC) [66] to assess CPTSDS. The items are answered with reference to the previous month that are answered on a 4-point Likert scale (from “never” = 0, to “very often” = 3). According to Elklit et al. [64], five items from the TSC and one item from the HTQ were used in the CPTSD item set to assess the CPTSD clusters (affective dysregulation, negative self-concept, and disturbances in relationships). The items representing PTSD and CPTSD are shown in Table 2. The reliability of the item set (α = 0.79) was satisfactory to good.

**Table 2:** Items representing PTSD symptoms and complex PTSD

Cluster	Test items
PTSD symptoms	HTQ 2. Feeling as though the event is happening again HTQ 3. Recurrent nightmares HTQ 6. Being jumpy or easily startled HTQ 9. Feeling on guard HTQ 11. Avoiding activities that remind you of the traumatic or hurtful event HTQ 15. Avoiding thought or feelings associated with the traumatic or hurtful events
Affect dysregulation	TSC 16. Temper outburst that you could not control
	TSC 14. Crying easily
Negative self-concept	TSC 28. Feelings of inferiority or insecurity
	TSC 29. Blaming yourself
Interpersonal problems	TSC 6. Feeling isolated from other people
	HTQ 27. Feeling that you have no one to rely upon

### Data analysis

Data analysis was firstly conducted using the IBM SPSS Statistics for Windows (version 28). Multiple Pearson correlation analyses were conducted to test bi-variate relationships between the study variables. Coefficients ranging between ± 0.50 and ± 1 indicate a strong correlation; coefficients ranging between ± 0.30 and ± 0.49 indicate a medium correlation; coefficients below ± 0.29 indicate a small correlation [67].

Multiple step mediation methodology, with a bootstrapped confidence interval for indirect effects, was conducted to test our hypothesis of serial mediation [68]. The final model included four outcome variables (PTSS, Affective dysregulation, Negative self-concept, and Disturbances in relationships), so that examination of this model was performed through Structural Equation Modeling (SEM). Specifically, the following was examined: (a) if both direct and indirect exposure to ACE were directly linked to PTSS and CPTSDS clusters; (b) if direct and indirect exposure to ACE were directly linked to MDM, NDM, and IDM; (c) if MDM, NDM, and IDM were directly linked to PTSS and CPTSDS clusters; (d) if direct and indirect exposure to ACE were indirectly linked to PTSS and CPTSDS clusters through MDM, NDM, and IDM.

Despite indirect exposure to ACE has not been associated with adverse consequences on individuals’ self-concept, the testimony of widespread economic precarity and community violence that characterizes India may have an impact on adolescents’ self-concept. For this reason, it was tested the direct path from indirect exposure to ACE to negative self-concept. Moreover, NDM were not considered as mediators of the link between exposure to ACE with PTSS and CPTSDS clusters. However, some previous findings observed that higher exposure to ACE are linked to higher levels of NDM [47]. For this, reason NDM were introduced into the models.

Regarding missing values in the tested variables, cautious procedures were adopted during data collection (e.g., checking if participants did respond to all items). For this reason, there were no missing values in our data.

A SEM strategy using the AMOS software (Version 29) [68] and the Maximum Likelihood method was employed to test the serial mediation model [69]. The following criteria for models fit were adopted: (a) χ2 test value should be non-significant, (b) the comparative fit index (CFI), the normed fit index (NFI), and the Tucker Lewis Index (TLI) should be higher than 0.95; (c) the root mean square error of approximation (RMSEA) and the standardized root mean square residual (SRMR) should range from 0.00 to 0.08 [70].

All analyses included only participants who had undergone at least one traumatic event. To assess significance of indirect paths, a bootstrapped confidence interval for the ab indirect effect, employing Preacher and Hayes’ procedures [71], was adopted. A total of 5,000 bootstrapped samples were obtained to estimate indirect effects of each mediator. We computed bias corrected, accelerated 95% confidence intervals (CIs) to measure statistical significance for each mediator’s “ab” paths and the one-step mediation. A Confidence Interval that does not include zero reflects evidence of a significant indirect effect or significant mediation.

## Results

### Prevalence of adverse childhood experiences

As can be seen in Table 3, the most common event was indirect exposure to traffic accidents which was reported by more than half of the participants. Indirect and direct exposure to the death of someone close were reported by nearly half of the participants. Least prevalent was indirect exposure to other events, followed by direct exposure to rape, attempted suicide, divorce, and sexual abuse. The prevalence of indirect exposure to ACE was generally higher than direct exposure.

**Table 3:** Potential trauma events and life events according to direct and indirect exposure

Events	Direct exposure Count (%)	Indirect exposure Count (%)
Traffic accident	161 (39.2%)	214 (52.1%)
Other serious accidents	70 (17.0%)	132 (32.1%)
Physical assault	32 (7.8%)	44 (10.7%)
Rape	5 (1.2%)	15 (3.6%)
Witnessed other people injured or killed	76 (18.5%)	88 (21.4%)
Came close to being injured or killed	75 (18.2%)	62 (15.1%)
Threats of violence	43 (10.5%)	50 (12.2%)
Near-drowning	35 (8.5%)	34 (8.3%)
Attempted suicide	10 (2.4%)	42 (10.2%)
Robbery/theft	45 (10.9%)	101 (24.6%)
Pregnancy /abortion	14 (3.4%)	79 (19.2%)
Serious illness	113 (27.5%)	153 (37.5%)
Death of someone close	170 (41.4%)	176 (43.1%)
Divorce	10 (2.4%)	28 (6.8%)
Sexual abuse	11 (2.7%)	16 (3.9%)
Physical abuse	25 (6.1%)	31 (7.5%)
Severe childhood neglect	18 (4.4%)	41 (10.0%)
Humiliation or persecution (bullying)	48 (11.7%)	48 (11.7%)
Absence of a parent	34 (8.3%)	54 (13.1%)
Other events	19 (4.6%)	3 (0.7%)

Only 23 participants (5.6%), 13 females and 10 males, did not report exposure to at least one ACE (direct or indirect exposure). These participants were excluded from subsequent analyses. The average number of total exposure to ACE per participant was 5.9 (SD = 4.8; range 0–24). The average number of direct exposure to ACE per participant was 2.5 (SD = 2.5; range 0–14) and the average number of indirect exposure to ACE per participant was 3.5 (SD = 3.3; range 0–19).

### Intercorrelations between study variables

As can be seen in Table 4, direct exposure to ACE had a medium positive correlation with indirect exposure to ACE, and a weak positive correlation with NDM, IDM and all clinical symptoms. The indirect exposure to ACE was weakly positively linked to IDM and clinical symptoms. MDM had a medium positive association with NDM, a weak positive correlation with IDM, and weak negative association with both Affective dysregulation and Disturbances in relationships. NDM presented weak positive associations with IDM, PTSS and Negative self-concept. IDM were weakly positively associated with Affective dysregulation, and presented a medium positive association with PTSS, Negative self-concept and Disturbances in relationships. Affective dysregulation was weakly positively linked to Negative self-concept and presented a medium positive correlation with Negative self-concept and Disturbances in relationships. Negative self-concept and Disturbances in relationships were weakly positively correlated. All the remaining associations were non-significant.

**Table 4:** Correlation matrix of study variables

Variables	1	2	3	4	5	6	7	8	9
1. Direct exposure to ACE	-	0.33***	− 0.01	0.15**	0.26**	0.30***	0.27***	0.22***	0.20***
2. Indirect exposure to ACE			0.07	0.09	0.13*	0.19***	0.14**	0.19***	0.12*
3. MDM			-	0.34***	0.17**	− 0.09	− 0.13*	− 0.01	− 0.14**
4. NDM				-	0.24***	0.16**	0.08	0.14**	0.07
5. IDM					-	0.43***	0.30***	0.34***	0.40***
6. PTSS						-	0.42***	0.36***	0.53***
7. Affective dysregulation							-	0.30***	0.44***
8. Negative self-concept								-	0.35***
9. Disturbances in relationships									-

*Note*. ACE = Adverse Childhood Experiences; MDM = Mature Defense Mechanisms; NDM = Neurotic Defense Mechanisms; IDM = Immature Defense Mechanisms; PTSS = PTSD symptoms. *p < .05. **p < .01. ***p < .001

### Analysis of serial mediation

In Model 1, it was tested the direct paths from direct and indirect exposure to ACE to PTSS and CPTSDS clusters. This model fits the observed data well (*χ*^2^ (1) = 1.33, *p* = .25; NFI = 1.0; CFI = 1.0; TLI = 0.99; RMSEA = 0.03; SMSR = 0.01). Higher direct exposure to ACE was significantly associated with higher levels of PTSS (*b* = 0.31, *p* < .01, 95% CI, 0.16, 0.46), Affective dysregulation (*b* = 0.14, *p* < .01, 95% CI, 0.09, 0.19), Negative self-concept (*b* = 0.09, *p* < .01, 95% CI, 0.04, 0.14), and Disturbances in relationships (*b* = 0.10, *p* < .01, 95% CI, 0.04, 0.16). Higher indirect exposure to ACE was significantly associated with higher levels of PTSS (*b* = 0.13, *p* < .05, 95% CI, 0.02, 0.24) and Negative self-concept (*b* = 0.05, *p* < .05, 95% CI, 0.02, 0.08). Indirect exposure to ACE was not significantly associated with levels of Affective dysregulation (*b* = 0.03, *p* = .23, 95% CI, − 0.01, 0.07) and Disturbances in relationships (*b* = 0.02, *p* = .25, 95% CI, − 0.02, 0.06).

In Model 2, it was tested the direct paths from direct and indirect exposure to ACE had direct paths to DM. This model fits the observed data well (*χ*^2^ (1) = 0.32, *p* = .47; NFI = 1.0; CFI = 1.0; TLI = 1.0; RMSEA = 0.0; SMSR = 0.01). Higher direct exposure to ACE was significantly associated with higher levels of NDM (b = 0.55, p < .05, 95% CI, 0.13, 0.97) and IDM (b = 2.04, p < .01, 95% CI, 1.17, 2.91). However, direct exposure to ACE was not associated with levels of MDM (*b* = − 0.12, *p* = .57, 95% CI, − 0.52, 0.38). Likewise, indirect exposure to ACE was not associated with levels of MDM (*b* = 0.15, *p* = .15, 95% CI, − 0.15, 0.45), NDM (*b* = 0.23, *p* = .13, 95% CI, − 0.08, 0.54), and IDM (*b* = 0.33, *p* =-.33, 95% CI, − 0.33, 0.98).

In Model 3, it was tested if the mediators, i.e., DM, had direct paths to clinical symptoms. The model fits the observed data well (*χ*^*2*^ (1) = 0.08, *p* = .38; NFI = 1.0; CFI = 1.0; TLI = 1.0; RMSEA = 0.0; SMSR = 0.01). Higher levels of MDM were significantly associated with lower levels of PTSS (*b* = − 0.07, *p* < .01, 95% CI, − 0.11, − 0.03), Affective dysregulation (*b* = − 0.03, *p* < .05, 95% CI, − 0.04, − 0.02), and Disturbances in relationships (*b* = − 0.03, *p* < .05, 95% CI, − 0.04, − 0.02). However, MDM was not associated with levels of Negative self-concept (b = − 0.01, p = .06, 95% CI, − 0.02, 0.0). Higher levels of NDM were only significantly associated with higher levels of PTSS (b = 0.07, p < .05, 95% CI, 0.03, 0.11). Levels of NDM were not associated with Affective dysregulation (*b* = 0.01, *p* = .17, 95% CI, 0.0, 0.02), Negative self-concept (*b* = 0.01, *p* = .09, 95% CI, 0.0, 0.02), and Disturbances in relationships (*b* = 0.01, *p* = .38, 95% CI, 0.0, 0.02). Higher levels of IDM were significantly associated with higher levels of PTSS (*b* = 0.05, *p* < .01, 95% CI, 0.03, 0.07), Affective dysregulation (*b* = 0.02, *p* < .05, 95% CI, 0.01, 0.03), Negative self-concept (*b* = 0.02, *p* < .05, 95% CI, 0.01, 0.03), and Disturbances in relationships (*b* = 0.03, *p* < .05, 95% CI, 0.02, 0.04).

Finally, Model 4 tested direct and indirect paths from direct and indirect exposure to ACE to PTSS and CPTSDS clusters, and one-step indirect paths through MDM, NDM, and IDM. Unstandardized coefficients and bootstrap solutions are presented in Table 5. The observed data fit the mediational model well (*χ*^2^ (1) = 0.32, *p* = .57; NFI=. 1.0; CFI = 1.0; TLI = 1.0; RMSEA = 0.0; SMSR = 0.01).

Although the magnitude was attenuated, the direct paths from direct exposure to ACE to PTSS and Affective dysregulation remained significant when the model included the mediators. However, the direct path from direct exposure to ACE to Negative self-concept and Disturbances in relationships were no longer significant. The direct paths from indirect exposure to ACE to PTSS and Negative self-concept remained significant when the mediators were included in the model. It was also observed that the indirect effects from direct exposure to ACE to PTSS and CPTSD symptoms clusters trough IDM were significant. Specifically, the results indicated that higher direct exposure to ACE was significantly associated with higher levels of IDM, which in turn were associated with higher levels of PTSS and all CPTSD symptom clusters. It was also observed that the indirect effects from direct exposure to ACE to PTSS trough NDM were significant, that is, higher exposure to ACE was significantly associated with higher levels of NDM, which in turn were related to higher levels of PTSS. It also observed that higher levels of MDM were related to lower levels of PTSS, Affective dysregulation, and Disturbances in relationships.

**Table 5:** Bootstrapped point estimate for direct and indirect effects for predicting PTSD and complex PTSD symptoms

	Point estimate	SE	BCa 95% CI (lower, upper)
PTSD symptoms			
Direct effect of direct exposure	0.17	0.09	(0.02, 0.32)*
Direct effect of indirect exposure	0.13	0.06	(0.02, 0.24)*
Indirect effect of direct exposure via MDM	− 0.12	0.21	(-0.52, 0.28)
Indirect effect of indirect exposure via MDM	0.23	0.15	(-0.07, 0.53)
Indirect effect of direct exposure via NDM	0.55	0.21	(0.13, 0.97)**
Indirect effect of indirect exposure via NDM	0.14	0.16	(-0.17, 0.45)
Indirect effect of direct exposure via IDM	2.04	0.44	(1.17, 2.91)***
Indirect effect of indirect exposure via IDM	0.33	0.33	(-0.32, 0.98)
Affective dysregulation			
Direct effect of direct exposure	0.09	0.03	(0.04, 0.14)***
Direct effect of indirect exposure	0.02	0.02	(-0.02, 0.06)
Indirect effect of direct exposure via MDM	0.08	0.05	(-0.01, 0.17)
Indirect effect of indirect exposure via MDM	− 0.01	0.03	(-0.06, 0.04)
Indirect effect of direct exposure via NDM	0.01	0.01	(-0.01, 0.03)
Indirect effect of indirect exposure via NDM	0.05	0.08	(-0.08, 0.24)
Indirect effect of direct exposure via IDM	1.43	0.23	(0.99, 1.87)***
Indirect effect of indirect exposure via IDM	0.02	0.02	(-0.02, 0.06)
Negative self-concept			
Direct effect of direct exposure	0.04	0.03	(-0.01, 0.09)
Direct effect of indirect exposure	0.04	0.02	(0.01, 0.07)*
Indirect effect of direct exposure via MDM	0.09	0.17	(-0.25, 0.42)
Indirect effect of indirect exposure via MDM	0.08	0.06	(-0.05, 0.21)
Indirect effect of direct exposure via NDM	0.04	0.06	(-0.08, 0.16)
Indirect effect of indirect exposure via NDM	0.01	0.01	(0.00, 0.02)
Indirect effect of direct exposure via IDM	0.09	0.01	(0.08, 0.10)***
Indirect effect of indirect exposure via IDM	0.02	0.03	(-0.03, 0.07)
Disturbances in relationships			
Direct effect of direct exposure	0.04	0.03	(-0.01, 0.09)
Direct effect of indirect exposure	0.03	0.02	(-0.01, 0.07)
Indirect effect of direct exposure via MDM	0.07	0.06	(-0.05, 0.19)
Indirect effect of indirect exposure via MDM	0.03	0.05	(-0.07, 0.13)
Indirect effect of direct exposure via NDM	0.01	0.01	(0.00, 0.02)
Indirect effect of indirect exposure via NDM	0.01	0.02	(-0.02, 0.04)
Indirect effect of direct exposure via IDM	0.40	0.14	(0.12, 0.68)***
Indirect effect of indirect exposure via IDM	0.06	0.04	(-0.02, 0.14)

*Note*. MDM = Mature Defense Mechanisms; NDM = Neurotic Defense Mechanisms; IDM = Immature Defense Mechanisms; BCa = bias corrected and accelerated; CI = confidence intervals; Confidence intervals that do not include 0 (null association) are significant. *p < .05. **p < .01. ***p < .001

**Fig. 1:**
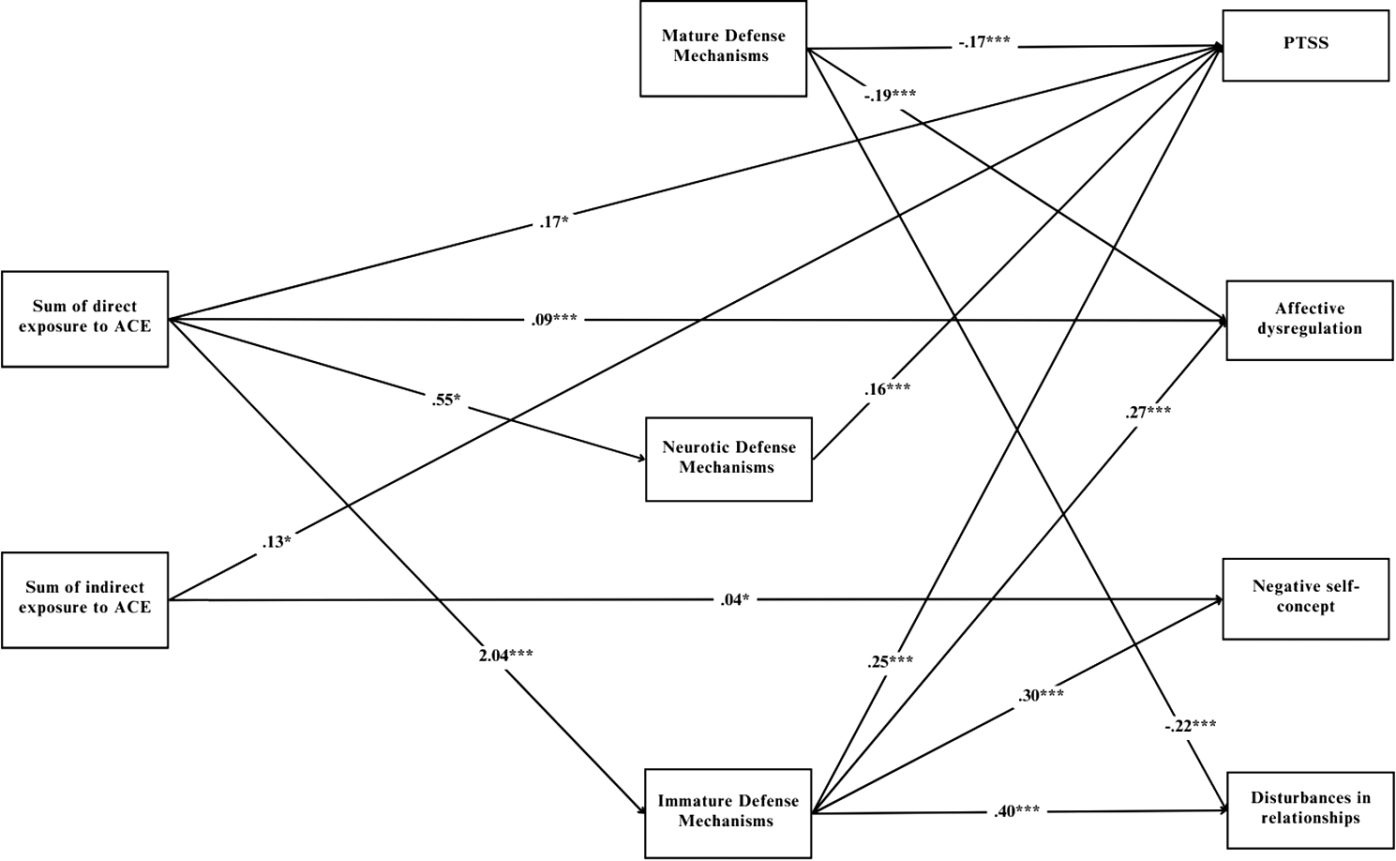
A serial mediational integrated model for PTSD and CPTSD symptoms by defense mechanisms

After omitting non-significant paths (i.e., direct exposure to ACE → MDM; indirect exposure to ACE → MDM; direct exposure to ACE → Negative self-concept; direct exposure to ACE → Disturbances in relationships; indirect exposure to ACE → Affect dysregulation; indirect exposure to ACE → Disturbances in relationships; indirect exposure to ACE → NDM; indirect exposure to ACE → IDM; NDM → Affective dysregulation; NDM → Negative self-concept; NDM → Disturbance in relationships) our final model fit the observed data well (χ^2^ (12) = 16.63, *p* = .16; NFI=. 0.97; CFI = 0.99; TLI = 0.98; RMSEA = 0.03; SMSR = 0.04). Unstandardized results of this model are presented in Fig. 1.

Rectangles indicate measured variables. Unidirectional arrows depict hypothesized directional links. Standardized maximum likelihood parameters are used. Bold line estimates are statistically significant. N = 388; *p < .05, ***p < .001.

## Discussion

The main goal of the current study was to analyze the effect of direct and indirect exposure to ACE on both PTSS and CPTSDS clusters (affective dysregulation, negative self-concept, and disturbances in relationships) through the mediation of DM in a sample of Indian adolescents. To the best of our knowledge, this is a pioneer study on researching the joint association between these variables in this population. Our main results indicated that both IDM and NDM mediated the association between direct exposure to ACE with PTSS. Serial multiple mediation model results indicated that higher direct exposure to ACE was associated with higher levels of both IDM and NDM, which in turn were associated with higher PTSS severity. It was also observed that IDM were mediators of the relationship between direct exposure to ACE and CPTSDS clusters. Specifically, higher direct exposure to ACE was linked to higher levels of IDM which were then associated with higher levels of all CPTSDS clusters.

The current findings support prior research, indicating that Indian adolescents are especially vulnerable for being exposed to multiple ACE, both directly and indirectly [16–18]. Even though our sample were from a middle and upper-class background, our findings suggest that they still present increased risk for exposure to different types of ACE. This could be explained by the psychological and socioeconomical context lived in India, characterized by cultural beliefs in harsh discipline, gender-based inequalities and constant threat of violence (e.g., terrorist attacks and/or riots) [16, 17]. These combined factors could potentiate the likelihood for exposure to multiple types of ACE, namely among adolescents. Our hypotheses will be discussed in turn.

Our first hypothesis was that higher direct exposure to ACE was associated with higher levels of PTSS and CPTSD symptoms. This hypothesis was partially supported. It was observed a significant positive association between higher direct exposure to ACE and higher levels of PTSS and affective dysregulation. It seems that multiple direct exposure to ACE is directly associated with higher PTSS severity [7, 8] and difficulties in the capacity for self-regulation [33]. However, even though a direct association between direct exposure to ACE and negative self-concept and disturbances in relationships was found when direct paths were tested, these were no longer significant when the mediators were included in the model. This suggests that the impact of exposure to ACE on adolescents’ self-concept and ability to sustain relationships and being close to others is influenced by their implicit emotion regulation strategies, namely DM [34].

Our second hypothesis was that higher indirect exposure to ACE was associated with higher levels of PTSS. This was fully supported by our results. The current results replicate Zimmerman and Posick’s findings [6] on the association between ACE and PTSS, specifically the attenuated magnitude of the direct path from indirect exposure to PTSS when compared to the direct path from direct exposure. However, an unexpected finding was found. There was a positive association between higher indirect exposure to ACE and CPTSDS of negative self-concept. The confrontation with the context of psychological and socioeconomical adversity typical of India can have a negative impact on adolescents’ self-perception, self-efficacy, and their beliefs of personal mastery over their future [72].

Our third hypothesis was that both higher direct and indirect exposure to ACE were associated with higher levels of IDM and NDM and, conversely, lower levels of MDM. This was partially supported. Direct exposure to ACE was only associated with IDM and NDM. This is corroborated by prior findings who also found higher levels of both IDM and NDM associated with higher exposure to ACE among adolescents [8, 47, 48]. The Indian societal problems, many of them grounded in family and upbringing, could result in a regression to survival-serving defenses to cope with this context of multiple adversity [45, 46]. The lack of association between exposure to ACE with MDM indicates that a context of adversity does not result in decreased use of MDM, but rather on greater use of maladaptive defenses [73].

Our fourth hypothesis was that higher levels of IDM were associated with higher levels of PTSS and CPTSDS. This was fully supported by our findings. This goes in accordance with previous studies that observed the relationship between IDM and both PTSS [39, 56] and CPTSDS [58]. It seems that adolescents that predominantly use DM as an attempt to distort or refuse the recognition of the context of repeated adversity present higher risk of psychological distress [8, 56]. To our current knowledge, our paper is the first to date in exploring the relationship between DM and CPTSDS in adolescents.

Our fifth hypothesis was that higher levels of MDM were associated with lower levels of PTSS and CPTSDS. This was only partially supported. We found a negative association between higher levels of MDM and PTSS, but solely on two clusters of CPTSDS (affective dysregulation and disturbances in relationships). These results highlight the potential protective role of MDM against psychological distress following exposure to multiple types of ACE [14]. Our findings support prior studies that found no association between MDM and self-perception, self-efficacy, and adolescents’ beliefs about the future [72, 73].

Another unexpected finding was that higher levels of NDM were associated with higher levels of PTSS. These current results follow some previous findings on the role of NDM as a vulnerability factor for PTSS among adolescents [74]. The current findings suggest that higher use of NDM reflect an inhibitory mechanism to ward off intensive and negative emotions from awareness [75]. However, individuals who more frequently use NDM may find it hard to suppress their thoughts possibly triggering intrusive ideas and feeling, this way perpetuating the reexperience of the trauma and an enduring sense of threat [76].

Our sixth hypothesis was that direct and indirect exposure to multiple ACE were associated with higher levels of IDM, that will consequently relate to higher levels of PTSS and CPTSDS. This was partially supported by our results since IDM only mediated the association between higher levels of IDM with PTSS and CPTSDS. This effect was also found in a recent study that observed that IDM mediated the association between direct exposure and ACE with psychological symptoms in adolescents [8]. These findings suggest that adolescents who already resorted primarily to IDM, will more frequently use them in a context of polytraumatization. However, they could conversely be aggravating both their PTSS and CPTSDS [14, 58, 77].

Another unexpected result was found. It was observed that NDM mediated the association between direct exposure to ACE and PTSS. Since NDM seek to prevent the awareness of adverse feelings and thoughts, it seems that adolescents that more frequently use them as protective mechanisms against polytraumatization, actually become more vulnerable to experiencing intrusive memories and reexperiencing the traumatic event [73, 76]. Future studies should aim to better comprehend the existing associations between NDM and psychological distress in other samples of polytraumatized adolescents.

The current study has some limitations. First, the cross-sectional design precludes causal inferences, since inferring causality from serial mediation analyses is not feasible. Second, all data were collected based on self-report measures. Third, memory issues could bias the report of ACE, since it was collected retrospectively. Fourth, we could not identify the possibility of reoccurrence of the traumatic events. Fifth, even though this study used a validated measure to assess DM, future researchers should recur to alternative measures that focus on automatic and unconscious psychological processes. Seventh, the data were collected in 2012.

## Conclusions

Despite the limitations described, this study still provides valuable insight into the phenomenon of the effect of polytraumatization on adolescents’ mental health. The results found in this study highlight the vulnerability in Indian adolescents when it comes to multiple exposure to ACE. Clinicians working side by side with adolescents should assess not only potentially traumatic direct events, but also indirect exposure to adversity. Since being exposed to one ACE highly increase the probability to face new and different other traumatic events, it is recommended that clinicians pay attention to the adolescent’s entire traumatic history instead of focusing only on the most perceived traumatic event. We suggest that the clinicians’ focus on PTSD diagnosis should be complemented with a parallel assessment of CPTSD, especially in adolescents living in adverse contexts of community violence and disadvantaged living conditions and/or that come from “risky families” [78]. The influence of the social environment in which the child is integrated will also have an impact on their life (e.g., peer victimization and/or isolation) possibly contributing to its vulnerability and putting them and their families at risk. It is crucial that clinicians start interpreting and analyzing ACE from a holistic, almost systemic approach: child, family, and environment, mutually inseparable and, simultaneously, acting both as possible sources of stress and support. Finally, these results also highlight the important role of DM (both IDM and NDM) as psychological processes intervening in the link between polytraumatization and psychological symptoms. It is therefore recommended that clinicians assess and analyze the predominant DM in use, especially those that hinder self-regulation and emotion regulation capabilities to cope with traumatic experiences. Intervention should focus on the promotion of more adaptative and healthy DM when confronted with multiple exposure to ACE.

## Data Availability

The data that support the findings of this study are available on request from the corresponding author FERRAJÃO. The data are not publicly available due to them containing information that could compromise research participant privacy.

## Abbreviations

ACE: Adverse Childhood Experiences
CPTSDS: Complex Post-Traumatic Stress Disorder symptoms
DM: Defense mechanisms
IDM: Immature defense mechanisms
MDM: Mature defense mechanisms
NDM: Neurotic defense mechanisms
PTSS: Post-Traumatic Stress Disorder symptoms
SEM: Structural Equation Modeling

## References

[1] Finkelhor D, Shattuck A, Turner H, Hamby S (2015). A revised inventory of adverse childhood experiences. Child Abuse Negl.

[2] Kalmakis KA, Chandler GE (2014). Adverse childhood experiences: towards a clear conceptual meaning. J Adv Nurs.

[3] Struck S, Stewart-Tufescu A, Asmundson AJN, Asmundson GGJ, Afifi TO (2021). Adverse childhood experiences (ACEs) research: a bibliometric analysis of publication trends over the first 20 years. Child Abuse Negl.

[4] Dube SR, Felitti VJ, Dong M, Chapman DP, Giles WH, Anda RF (2003). Childhood abuse, neglect, and household dysfunction and the risk of illicit drug use: the adverse childhood experiences study. Pediatrics.

[5] Edwards VJ, Holden GW, Felitti VJ, Anda RF (2003). Relationship between multiple forms of childhood maltreatment and adult mental health in community respondents: results from the adverse childhood experiences study. Am J Psychiatry.

[6] Zimmerman GM, Posick C (2016). Risk factors for and behavioral consequences of direct versus indirect exposure to Violence. Am J Public Health.

[7] Bajo M, Blanco A, Stavraki M (2018). Post-traumatic cognitions and quality of life in Terrorism victims: the role of well-being in indirect versus direct exposure. Health Qual Life Outcomes.

[8] Ferrajão P, Dias J, Elklit A (2018). Defense mechanisms mediate associations between exposure to adverse childhood experiences and anxiety and depression in Kenyan adolescents. Traumatology.

[9] Contractor AA, Caldas S, Fletcher S, Shea MT, Armour C (2018). Empirically derived lifespan polytraumatization typologies: a systematic review. J Clin Psychol.

[10] Anastas JW, Payne NA, Ghuman SA (2021). Adverse childhood experiences and complex post-traumatic stress in pregnant teens: a pilot study. Matern Child Health J.

[11] Briere J, Agee E, Dietrich A (2016). Cumulative trauma and current posttraumatic stress disorder status in general population and inmate samples. Psychol Trauma.

[12] Alvarez BD, Razente DM, Lacerda DA, Lother NS, VON-Bahten LC, Stahlschmidt CM (2016). Analysis of the revised trauma score (RTS) in 200 victims of different trauma mechanisms. Rev Col Bras Cir.

[13] Breslau N, Koenen KC, Luo Z (2014). Childhood maltreatment, juvenile disorders and adult post-traumatic stress disorder: a prospective investigation. Psychol Med.

[14] Waikamp V, Serralta FB, Ramos-Lima LF, Zatti C, Freitas LHM (2021). Relationship between childhood trauma, parental bonding, and defensive styles and psychiatric symptoms in adult life. Trends Psychiatry Psychother.

[15] Nooner KB, Linares LO, Batinjane J, Kramer RA, Silva R, Cloitre M (2012). Factors related to posttraumatic stress disorder in adolescence. Trauma Violence Abuse.

[16] Deb, Deb S, Banu PR, Thomas S, Vardhan RV, Rao PT, Khawaja N et al. Depression among Indian university students and its association with perceived university academic environment, living arrangements and personal issues. Asian J Psychiatr. 2016; 10.1016/j.ajp.2016.07.010. 10.1016/j.ajp.2016.07.010 27969066

[17] Rasmussen DJ, Karsberg S, Karstoft KI, Elklit A (2013). Victimization and PTSD in an Indian youth sample from Pune City. Open Journ of Epid.

[18] Kacker L, Varadan S, Kumar P (2007). Study on Child Abuse: India 2007.

[19] Kumar M (2007). A journey into the bleeding city: following the footprints of the rubble of Riot and Violence of Earthquake in Gujarat, India. Psychol and Develop Soci.

[20] Kar N, Mohapatra PK, Nayak KC, Pattanaik P, Swain SP, Kar HC (2007). Post-traumatic stress disorder in children and adolescents one year after a super-cyclone in Orissa, India: exploring cross-cultural validity and vulnerability factors. BMC Psychiatry.

[21] Seethalakshmi R, Dhavale HS, Gawande S, Dewan M (2006). Psychiatric morbidity following motor vehicle crashes: a pilot study from India. J Psychiatr Pract.

[22] Varma D, Chandra PS, Thomas T, Carey MP (2007). Intimate partner Violence and sexual Coercion among pregnant women in India: relationship with depression and post-traumatic stress disorder. J Affect Disord.

[23] Cloitre M, Hyland P, Bisson JI (2019). ICD-11 Posttraumatic Stress Disorder and Complex Posttraumatic Stress Disorder in the United States: a Population-based study. J Trauma Stress.

[24] Messman-Moore TL, Bhuptani PH (2017). A review of the long-term impact of child maltreatment on posttraumatic stress disorder and its comorbidities: an emotion dysregulation perspective. Clin Psyc: Scienc Pract.

[25] Ho GWK, Karatzias T, Vallières F (2021). Complex PTSD symptoms mediate the association between childhood trauma and physical health problems. J Psychosom Res.

[26] Kampling H, Kruse J, Lampe A (2022). Epistemic trust and personality functioning mediate the association between adverse childhood experiences and posttraumatic stress disorder and complex posttraumatic stress disorder in adulthood. Front Psychiatry.

[27] World Health Organization. International statistical classification of diseases and related health problems.11th ed., 2019; https://icd.who.int/.

[28] Karatzias T, Shevlin M, Ford JD (2022). Childhood trauma, attachment orientation, and complex PTSD (CPTSD) symptoms in a clinical sample: implications for treatment. Dev Psychopathol.

[29] Guo T, Huang L, Hall DL (2021). The relationship between childhood adversities and complex posttraumatic stress symptoms: a multiple mediation model. Eur J Psychotraumatol.

[30] Fox BH, Perez N, Cass E, Baglivio MT, Epps N (2015). Trauma changes everything: examining the relationship between adverse childhood experiences and serious, violent and chronic juvenile offenders. Child Abuse Negl.

[31] Straussner SLA, Calnan AJ (2014). Trauma through the life cycle: a review of current literature. Clin Soc Work J.

[32] Karatzias T, Shevlin M, Fyvie C (2017). Evidence of distinct profiles of posttraumatic stress disorder (PTSD) and Complex Posttraumatic stress disorder (CPTSD) based on the new ICD-11 Trauma Questionnaire (ICD-TQ). J Affect Disord.

[33] Poole JC, Dobson KS, Pusch D (2018). Do adverse childhood experiences predict adult interpersonal difficulties? The role of emotion dysregulation. Child Abuse Negl.

[34] van Dijke A, Hopman JAB, Ford JD (2018). Affect dysregulation, psychoform dissociation, and adult relational fears mediate the relationship between childhood trauma and complex posttraumatic stress disorder Independent of the symptoms of borderline personality disorder. Eur J Psychotraumatol.

[35] Koole SL, Rothermund K (2011). I feel better but I don’t know why: the psychology of implicit emotion regulation. Cogn Emot.

[36] Gyurak A, Gross JJ, Etkin A (2011). Explicit and implicit emotion regulation: a dual-process framework. Cogn Emot.

[37] Schwager S, Rothermund K (2013). Counter-regulation triggered by emotions: positive/negative affective states elicit opposite valence biases in affective processing. Cogn Emot.

[38] Rice TR, Hoffman L (2014). Defense mechanisms and implicit emotion regulation: a comparison of a psychodynamic construct with one from contemporary neuroscience. J Am Psychoanal Assoc.

[39] Prout TA, Malone A, Rice T, Hoffman L (2019). Resilience, defense mechanisms, and implicit emotion regulation in psychodynamic child psychotherapy. J Cont Psychoth.

[40] Vaillant GE. Involuntary coping mechanisms: a psychodynamic perspective. Dialogues Clin Neurosci. 2011. 10.31887/DCNS.2011.13.2/gvaillant. 10.31887/DCNS.2011.13.2/gvaillant PMC3182012 22034454

[41] Bond M (2004). Empirical studies of defense style: relationships with psychopathology and change. Harv Rev Psychiatry.

[42] Wolmer L, Erez C, Toren P (2020). Defense style of children and adolescents: differences and ability to discriminate among clinical categories. J Nerv Ment Dis.

[43] Beresford TP (2012). Psychological adaptive mechanisms: Ego defense recognition in practice and research.

[44] Santana MRM, Zatti C, Spader ML (2017). Acute stress disorder and defense mechanisms: a study of Physical Trauma patients admitted to an emergency hospital. Trends Psychiatry Psychother.

[45] Perry JC, Metzger J, Sigal JJ (2015). Defensive functioning among women with Breast cancer and matched community controls. Psychiatry.

[46] Punamäki RL, Kanninen K, Qouta S, El-Sarraj E (2002). The role of psychological defences in moderating between trauma and post-traumatic symptoms among Palestinian men. Int J Psychology.

[47] Jun JY, Lee YJ, Lee SH, Yoo SY, Song J, Kim SJ (2015). Association between defense mechanisms and psychiatric symptoms in North Korean refugees. Compr Psychiatry.

[48] Korkmaz S, Kazgan A, Yıldız S (2020). Analysis of childhood traumas and defense styles in patients with tension headache. Prim Care Companion CNS Disord.

[49] Uygur ÖF, Tuman TC, Hurşitoğlu O (2022). The relationship between childhood trauma with defense styles in depression patients. Genel Tıp Dergisi.

[50] Cramer P, Blatt SJ, Ford RQ (1988). Defense mechanisms in the anaclitic and introjective personality configuration. J Consult Clin Psychol.

[51] Sandstrom MJ, Cramer P (2003). Defense mechanisms and psychological adjustment in childhood. J Nerv Ment Dis.

[52] Evans DW, Seaman JL (2000). Developmental aspects of psychological defenses: their relation to self-complexity, self-perception, and symptomatology in adolescents. Child Psychiatry Hum Dev.

[53] Muris P, Winands D, Horselenberg R (2003). Defense styles, personality traits, and psychopathological symptoms in nonclinical adolescents. J Nerv Ment Dis.

[54] Ruuttu T, Pelkonen M, Holi M (2006). Psychometric properties of the defense style questionnaire (DSQ-40) in adolescents. J Nerv Ment Dis.

[55] Diehl M, Chui H, Hay EL, Lumley MA, Grühn D, Labouvie-Vief G (2014). Change in coping and defense mechanisms across adulthood: longitudinal findings in a European American sample. Dev Psychol.

[56] Martino G, Caputo A, Bellone F, Quattropani MC, Vicario CM (2020). Going beyond the visible in type 2 Diabetes Mellitus: Defense mechanisms and their associations with depression and health-related quality of life. Front Psychol.

[57] Nickel R, Egle UT (2006). Psychological defense styles, childhood adversities and psychopathology in adulthood. Child Abuse Negl.

[58] Favaretto TC, Both LM, da Cruz Benetti SP, Freitas LHM (2022). Understanding the psychodynamic functioning of patients with PTSD and CPTSD: qualitative analysis from the OPD 2 interview. Psicol Reflex Crit.

[59] Finzi-Dottan R, Karu T (2006). From emotional abuse in childhood to psychopathology in adulthood: a path mediated by immature defense mechanisms and self-esteem. J Nerv Ment Dis.

[60] Fang S, Chung MC, Wang Y (2020). The impact of past trauma on psychological distress: the roles of defense mechanisms and alexithymia. Front Psychol.

[61] Bödvarsdóttir I, Elklit A (2007). Victimization and PTSD-like states in an Icelandic youth probability sample. BMC Psychiatry.

[62] Ferrajão P, Elklit A (2021). Attachment and social support mediate associations between polyvictimization and psychological distress in early Uganda and Kenya adolescents. Child Abuse Negl.

[63] Andrews G, Singh M, Bond M (1993). The Defense Style Questionnaire. J Nerv Ment Dis.

[64] Elklit A, Hyland P, Shevlin M (2014). Evidence of symptom profiles consistent with posttraumatic stress disorder and complex posttraumatic stress disorder in different trauma samples. Eur J Psychotraumatol.

[65] Mollica RF, Caspi-Yavin Y, Bollini P, Truong T, Tor S, Lavelle J (1992). The Harvard Trauma Questionnaire. Validating a cross-cultural instrument for measuring Torture, trauma, and posttraumatic stress disorder in indochinese refugees. J Nerv Ment Dis.

[66] Briere J, Runtz M (1989). The Trauma Symptom Checklist (TSC-33): early data on a new scale. Jl of Interpl Viol.

[67] Cohen J (1988). Statistical power analysis for the behavioral sciences.

[68] Hayes AF (2013). Introduction to mediation, moderation, and conditional process analysis: a regression-based approach.

[69] Arbuckle JL (2019). Amos Version 26.0.

[70] Hoyle RH, Smith GT (1994). Formulating clinical research hypotheses as structural equation models: a conceptual overview. J Consult Clin Psychol.

[71] Preacher KJ, Hayes AF (2008). Asymptotic and resampling strategies for assessing and comparing indirect effects in multiple mediator models. Behav Res Methods.

[72] Chung MC, AlQarni N, AlMazrouei M (2021). Posttraumatic stress disorder and psychiatric co-morbidity among Syrian refugees: the role of trauma exposure, trauma centrality, self-efficacy and emotional suppression. J Ment Health.

[73] Lenzo V, Barberis N, Cannavò M, Filastro A, Verrastro V, Quattropani MC (2020). The relationship between alexithymia, defense mechanisms, eating disorders, anxiety and depression. Riv Psichiatr.

[74] Shevlin M, Elklit A. (2008). Modelling the role of defense styles as mediating the relationship between trauma and posttraumatic stress severity in Danish adolescents. Irish J Psychol. 2008; 10.1080/03033910.2008.10446284.

[75] Colovic O, Lecic Tosevski D, Perunicic Mladenovic I, Milosavljevic M, Munjiza A (2016). Defense mechanisms in pure anxiety and pure depressive disorders. J Nerv Ment Dis.

[76] Hamza E, Helal A, Moustafa A, Emam M. (2020). The relationship between intrusive cognitive and defense mechanisms in healthy and clinical populations. *Humanit Soc Sci Rev*. 2020. 10.18510/hssr.2020.8191.

[77] Silverstein R (1996). Combat-related trauma as measured by ego developmental indices of defenses and identity achievement. J Genet Psychol.

[78] Repetti RL, Taylor SE, Seeman TE (2002). Risky families: family social environments and the mental and physical health of offspring. Psychol Bull.

